# Identification of Nitrogen, Phosphorus, and Potassium Deficiencies in Rice Based on Static Scanning Technology and Hierarchical Identification Method

**DOI:** 10.1371/journal.pone.0113200

**Published:** 2014-11-26

**Authors:** Lisu Chen, Lin Lin, Guangzhe Cai, Yuanyuan Sun, Tao Huang, Ke Wang, Jinsong Deng

**Affiliations:** Institute of Applied Remote Sensing & Information Technology, Zhejiang University, Hangzhou, Zhejiang, China; Zhejiang University, China

## Abstract

Establishing an accurate, fast, and operable method for diagnosing crop nutrition is very important for crop nutrient management. In this study, static scanning technology was used to collect images of a rice sample's fully expanded top three leaves and corresponding sheathes. From these images, 32 spectral and shape characteristic parameters were extracted using an RGB mean value function and using the Regionprops function in MATLAB. Hierarchical identification was used to identify NPK deficiencies. First, the normal samples and non-normal (NPK deficiencies) samples were identified. Then, N deficiency and PK deficiencies were identified. Finally, P deficiency and K deficiency were identified. In the identification of every hierarchy, SVFS was used to select the optimal characteristic set for different deficiencies in a targeted manner, and Fisher discriminant analysis was used to build the diagnosis model. In the first hierarchy, the selected characteristics were the leaf sheath R, leaf sheath G, leaf sheath B, leaf sheath length, leaf tip R, leaf tip G, leaf area and leaf G. In the second hierarchy, the selected characteristics were the leaf sheath G, leaf sheath B, white region of the leaf sheath, leaf B, and leaf G. In the third hierarchy the selected characteristics were the leaf G, leaf sheath length, leaf area/leaf length, leaf tip G, difference between the 2^nd^ and 3^rd^ leaf lengths, leaf sheath G, and leaf lightness. The results showed that the overall identification accuracies of NPK deficiencies were 86.15, 87.69, 90.00 and 89.23% for the four growth stages. Data from multiple years were used for validation, and the identification accuracies were 83.08, 83.08, 89.23 and 90.77%.

## Introduction

Rice shows obvious symptoms when suffering from nitrogen (N), phosphorus (P) and potassium (K) nutrition deficiencies, and these symptoms are the basis of rapid morphological diagnoses in the field. Morphological diagnoses require large amounts of experience. This method cannot be quantified and suffers from poor operability; farmers find it very difficult to expertly use this method. Diagnostic methods using digital imaging based on morphological diagnoses can dynamically and quantitatively extract information from the symptoms of nutrition stress, which can be used to automatically identify the nutrition status of rice.

**Figure 1:**
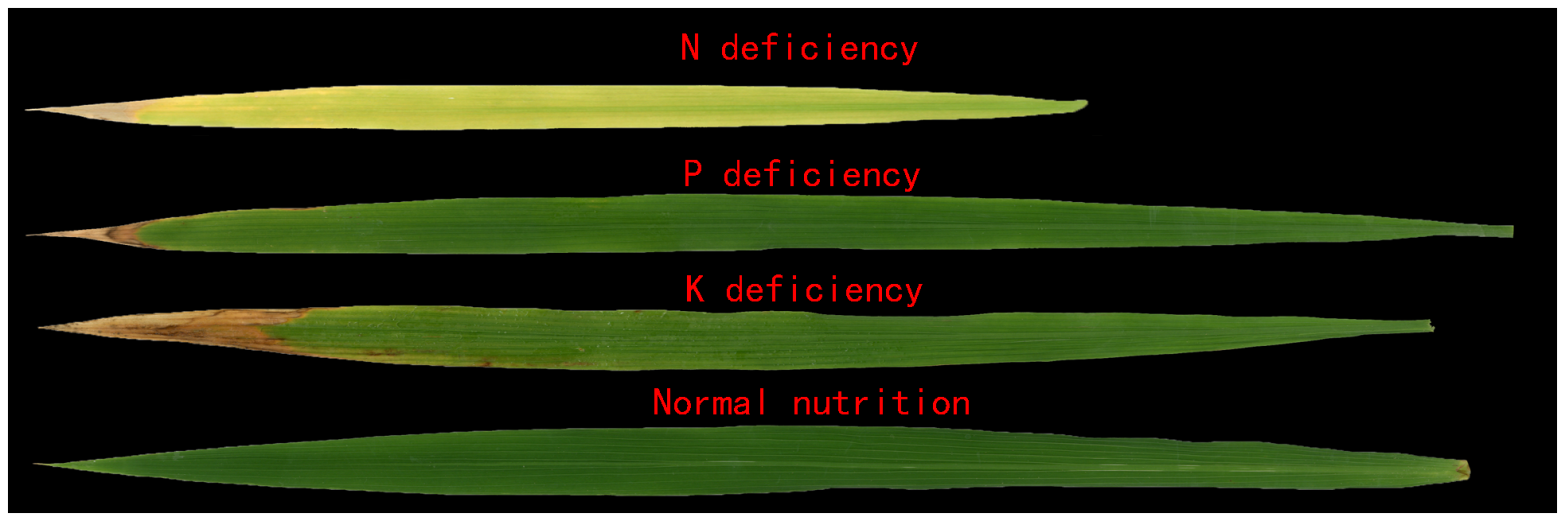
The different characteristics of rice leaves under NPK deficiencies.

**Figure 2:**
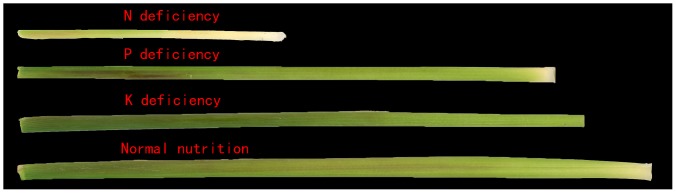
The different characteristics of rice sheaths under NPK deficiencies.

Rice with NPK deficiencies usually exhibits numerous symptoms. Under N deficiency, old leaves and sometimes all leaves become light green and chlorotic at the tip. Except for young leaves, which are greener, deficient leaves are narrow, short, erect, and lemon yellowish. Under P deficiency, leaves are narrow, short, very erect, and develop if the variety has a tendency to produce anthocyanin. Under K deficiency, dark-green plants with yellowish-brown leaf margins or dark-brown necrotic spots first appear on the tips of older leaves. Under severe K deficiency, leaf tips are yellowish brown. Older leaves change from yellow to brown [1]. Therefore, the color and shape of the leaf and sheath can indicate the plant nutrient and health status, which is closely related to the nutrition content (Fig. 1, Fig. 2).

Recently, the diagnosis of the nutrition status of rice has been based on hyperspectral characteristics, which are determined through the use of a hyper-spectrometer that measures the reflectance of rice canopies and leaves [2]–[9]. Although reflectance is different under different nutrition conditions, the reflectance curve has a similar waveform that makes it difficult to discriminate critical values. In addition, water stress, plant diseases and pests similarly influence the reflectance of canopies and leaves. Relying only on hyperspectral characteristics makes it difficult to build a single model to determine the nutrition status for practical analyses.

Rice under NPK deficiencies shows obvious symptoms in the color, shape and texture of the leaf. It is difficult to capture and quantify these micro-symptoms using a hyper-spectrometer. Because scanning is performed in a closed environment, which can reduce external disturbances during the image acquisition process, the accurate reproduction of the color and size of the sample can be ensured. Compared with a common digital camera, the scanned image does not include a complex background, multi-redundant information and image noise, which can reduce the error in the image analysis process. Thus, in this research, scanning is used to obtain a digital image to capture these symptoms. In previous studies, researchers mainly determined the plant nutrition status using information about the leaf [10]–[13]. When rice exhibits nutrition deficiency, the leaf sheath will also present specific symptoms [14]. Therefore, this study analyzed scanned images of rice leaves and sheaths to diagnose the nutrition status of various samples.

In this research, scanned images of rice leaves and sheaths under NPK deficiencies and normal nutrition levels were compared, and the differences in the rice leaf and sheath characteristics under the different nutrition conditions were analyzed. Fisher discriminant analysis was used to develop the rules and to construct a model for the identification of NPK deficiencies.

During the identification of the rice nutrition deficiencies, the standard identification process was to simultaneously identify the different types of deficiencies. When different deficiencies caused similar symptoms, it was easier to misjudge the deficiency type during the identification process. To improve the identification accuracy and to reduce misjudgments, hierarchical identification was used. With hierarchical identification, the identification process was formulated to identify particular nutrition deficiencies; therefore, a better identification could be performed.

### Ethics Statement

This study was designed to aid in the diagnosis of rice nutrition. All of the data in this study can be published and shared.

## Materials and Methods

### Experimental Design

The experiment was designed to study rice under different degrees of NPK deficiencies. Rice seeds (cultivar ZheYou-NO. 1) were pre-germinated in moist sand at 30°C for 3 days, and seedlings were individually transplanted 7 days after emergence into 5 L polyvinyl chloride (PVC) pots that contained clean, sieved, and thoroughly leached river sand to allow for precise nutrient control. In 2012 and 2013, the experiment was carried out in a greenhouse on the ZiJinGang campus of Zhejiang University (30°17′N, 120°05′E) in Hangzhou, China. The plants were grown under natural light conditions. The temperature of the greenhouse was maintained at 30°C/25°C (day/night), and the relative humidity was maintained at 50%. The nutrient solution was prepared with deionized water and contained 110.8 mg/L CaCl_2_, 405 mg/L MgSO_4_.7H_2_O, and 16.5 mg/L Na_2_SiO_3_.9H_2_O. The pots were arranged in 13 different levels of nutrition content (4 N level treatments, 4 P level treatments, 4 K level treatments, and normal nutrition treatment), 6 replication for each level, and 10 rice plants in each pot. A total of 13 treatment levels (NH_4_NO_3_ 0 mg/L, 28.60 mg/L, 57.20 mg/L, 85.70 mg/L; NaH_2_PO_4_.2H_2_O 0 mg/L, 12.60 mg/L, 25.20 mg/L, 37.80 mg/L; K_2_SO_4_ 0 mg/L, 22.30 mg/L, 44.70 mg/L, 67.00 mg/L; and normal nutrition (NH_4_NO_3_ 114.30 mg/L, NaH_2_PO_4_.2H_2_O 50.40 mg/L, K_2_SO_4_ 89.30 mg/L)) were produced via nutriculture (hydroponic) solutions and were added to different pots. The nutrient solutions in the pots were replaced every 14 days. Every 5 days, the pH of the nutrient solution in each pot was measured and adjusted to 5 using 1 mol/L NaOH.

### Acquisition images

The leaf samples were taken on August 4^th^, 18^th^, and 27^th^ and September 8^th^ of 2013. The top-three leaves for 10 rice plants with 13 nutrition levels, totaling 1560 samples and representing all growth stages, were collected. A total of 480 rice leaf and leaf sheath samples under 4 different N levels, 480 rice leaf and leaf sheath samples under 4 different P levels, 480 rice leaf and leaf sheath samples under 4 different K levels and 120 rice leaf and leaf sheath samples with normal nutrition levels were collected to build the diagnosis rules and the identification model for the NPK deficiencies. The rice leaf and leaf sheath samples collected on July 29^th^ and August 13^th^, 20^th^, and 31^st^ of 2012 were used to validate the model; these samples include leaves and leaf sheaths of plants grown under 4 different N levels (240 samples), 4 different P levels (240 samples), 4 different K levels (240 samples) and normal nutrition levels (60 samples).

All of the samples were analyzed in the laboratory. First, the leaves and sheaths were placed on a scanner (EPSON GT20000, Seiko Epson Corporation, Suwa, Nagano-ken, Japan) with a maximum scanning area of 11.7×17.0 inches and an R/G/B (the full color images consist of red (R), green (G), and blue (B) channels) and BK color CCD line sensor. The output image data were 16 bits per pixel per internal color and 1 to 8 bits per pixel per external color. The resolution was set to 300 dpi (dots per inch). The leaf area (cm^2^) can be calculated using the sum of all of the pixels within the range of the leaf multiplied by (2.54/300)^2^; the length (cm) equals the number of pixels in the vein multiplied by 2.54/300, and the width (cm) equals the number of pixels in the widest zone multiplied by 2.54/300.

### Feature selection

The R, G, B mean value function and the Regionprops function in MATLAB (MathWorks Inc., USA) were used to determine the leaf color characteristics (LR, LG, LB) and the shape characteristics (length (LL), width (LW), leaf area (LA), perimeter (LP), area/length (A/L), area/perimeter (A/P), eccentricity (EC), rectangularity (RE), area convexity (AC), circularity (CI), form factor (FF), and leaf sheath length (LSL)) (Table 1), which were used to identify the category of nutrition deficiency.

**Table 1:** The formulas and explanations of different characteristics.

Characteristic	Formula	Explanation
A/L	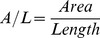	The ratio of leaf area to leaf length
A/P	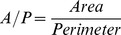	The ratio of leaf area to leaf perimeter
Eccentricity	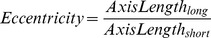	The ratio of leaf length to leaf width
Rectangularity	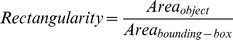	The ratio of leaf area to the area of the smallest box encasing the leaf
Area Convexity	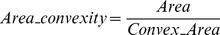	The ratio leaf area to the area of the convex hull of leaf
Circularity	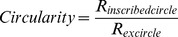	The ratio of an inscribed circle radius to a circumcircle radius
Form Factor	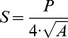	P and A are the leaf perimeter and area, respectively

In addition, considering that leaves with a phosphorus deficiency are dark green in color, lightness was added (LI) as a characteristic parameter in this study to increase identification accuracy [15].
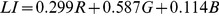
(1)


Because N, P, and K are very mobile within the plant and are translocated to young leaves from old, senescing leaves, the symptoms of rice suffering from NPK deficiencies often first appear at the tip of the leaf and subsequently spread to the entire leaf. Under N deficiency, leaves become light green at the tip, and the color then spreads to the entire leaf. Under PK deficiencies, the symptoms are similar, and the leaf tips become yellowish brown [1]. Therefore, the color of the leaf tip can be used to effectively identify symptoms of NPK deficiencies. In this research, the “thinning of color” characteristic was the mean color value for 1/5 of the leaf length from the tip and is expressed using the leaf tip R (LTR), leaf tip G (LTG), and leaf tip B (LTB).

In the rice-growing process, the color of the heart leaf is light compared to the next functional leaf. In the fully functional stage, when the second and third functional leaves have the darkest color and when the difference between the colors of neighboring leaves is small, rice grows well. In contrast, a larger difference means that the rice is suffering from a nutrition deficiency. At normal nutrition levels, the leaf length gradually increases with higher leaf positions; the top second or top third leaves are the longest. When the length of one leaf is shorter than or equal to the next leaf, the growth in the rice is restrained. Before the jointing stage, every leaf sheath is densely distributed on the tillering node, and the increased length of the leaf sheath reflects the leaf spacing which is the distance between neighboring leaves. The leaf spacing increases as the leaf grows. When the rice plant suffers from a nutrition deficiency, the spacing between leaves shortens. In line with this mechanism, this research added 8 parameters: the difference between the 2^nd^ and 3^rd^ leaf lengths (L23), the ratio of the 1^st^ to 3^rd^ leaf lengths (L1/3), the ratio of the leaf and leaf sheath lengths (L/LS), the difference between the 2^nd^ and 3^rd^ leaf R (R23), the difference between the 2^nd^ and 3^rd^ leaf G (G23), the difference between the 2^nd^ and 3^rd^ leaf B (B23), the leaf spacing of the 1^st^ and 2^nd^ leaves (LS12), the leaf spacing of the 2^nd^ and 3^rd^ leaves (LS23), and the difference in leaf spacing (LS12–LS23). In total, 28 color and shape parameters were extracted from the scanned images of the leaf and sheath (Table 2).

**Table 2:** Characteristic parameters of the scanned images.

NO.	Parameter	No.	Parameter	No.	Parameter	No.	Parameter
1	LR	8	EC	15	L/LS	22	B23
2	LG	9	RE	16	A/L	23	LS12
3	LB	10	AC	17	A/P	24	LS23
4	LL	11	CI	18	L23	25	LS12–LS23
5	LW	12	FF	19	L1/3	26	LTR
6	LA	13	LI	20	R23	27	LTG
7	LP	14	LSL	21	G23	28	LTB

**Figure 3:**
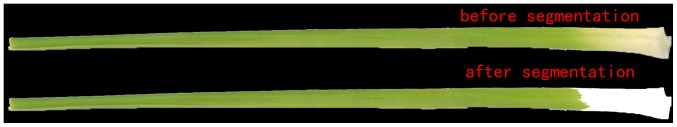
The segmentation of the rice sheath image.

Using the R, G, and B mean value function in MATLAB, the spectral characteristics of the leaf sheaths (LSR, LSG, LSB) under NPK stress and under normal nutrition levels were determined. Using the magic wand tool in Adobe Photoshop CS5, a segment in the white region of the leaf sheath (WRA) resulting from nitrogen stress was selected, and its area was calculated using the Regionprops function in MATLAB (Fig. 3). The four additional parameters for leaf sheath are shown in Table 3.

**Table 3:** Additional color features of the rice sheath.

No.	Parameter	No.	Parameter
29	LSR	31	LSB
30	LSG	32	WRA

**Figure 4:**
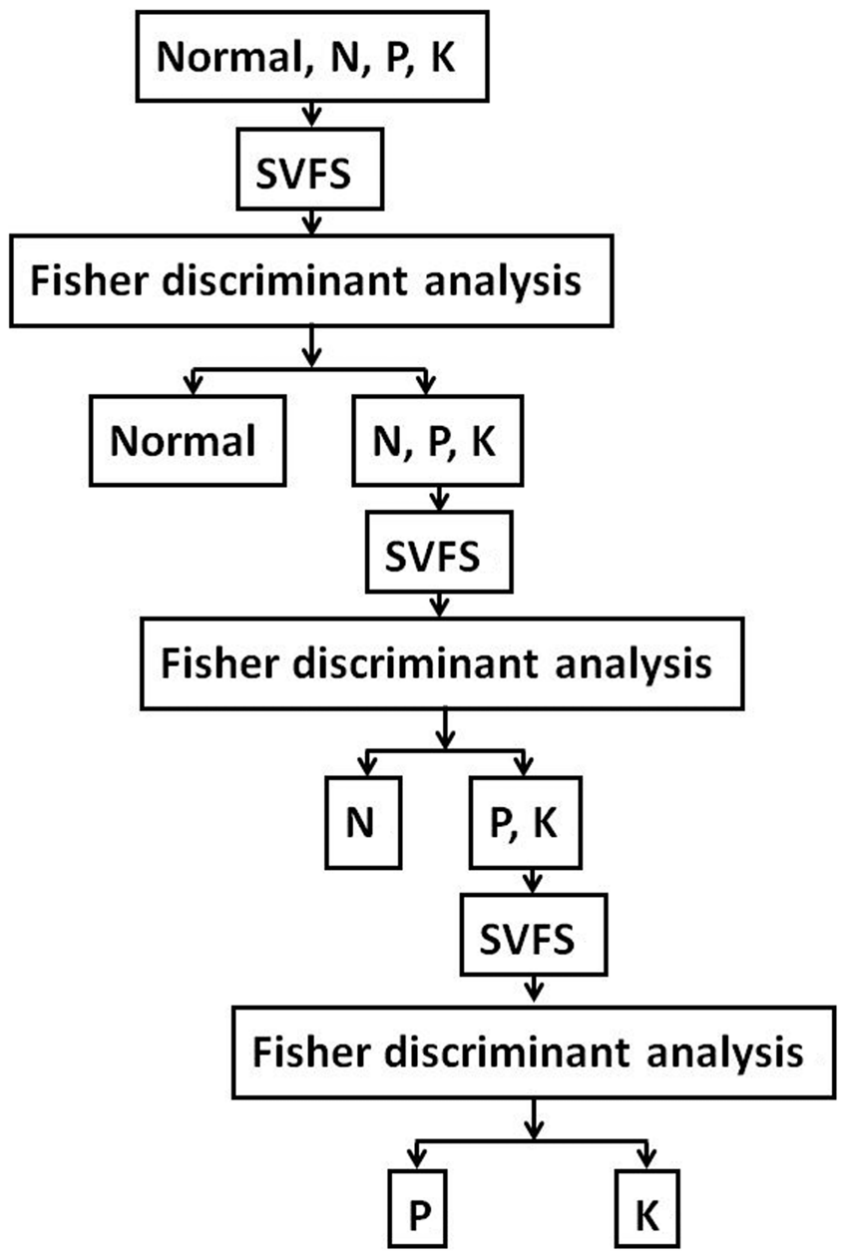
Hierarchical identification of the different nutrition deficiencies.

As shown in Fig. 4, 4 newly added color parameters of the leaf sheath showed obvious differences under the different nutrition conditions in the four growth stages.

### Research method

In this research hierarchical identification was used to identify deficiency category. In every hierarchy the selected characteristics were different, then using the characteristics selected by SVFS to establish rule and model of identification with Fisher discriminant analysis. In process the 1^st^ rule was established for the identification of normal and non-normal (NPK nutrition deficiency) states. According to the identification results, the non-normal samples were included in the second identification hierarchy. In the second hierarchy, the 2^nd^ rule was established for the identification of N and PK nutrition deficiencies. After removing the samples identified as having N nutrition deficiencies, the samples with PK nutrition deficiencies were included in the third identification hierarchy. The 3^rd^ rule was established for the identification of P and K nutrition deficiencies (Fig. 4). Finally, the samples with the correct identification of every nutrition deficiency in all of the identification processes were counted to calculate the identification accuracy.

**Figure 5:**
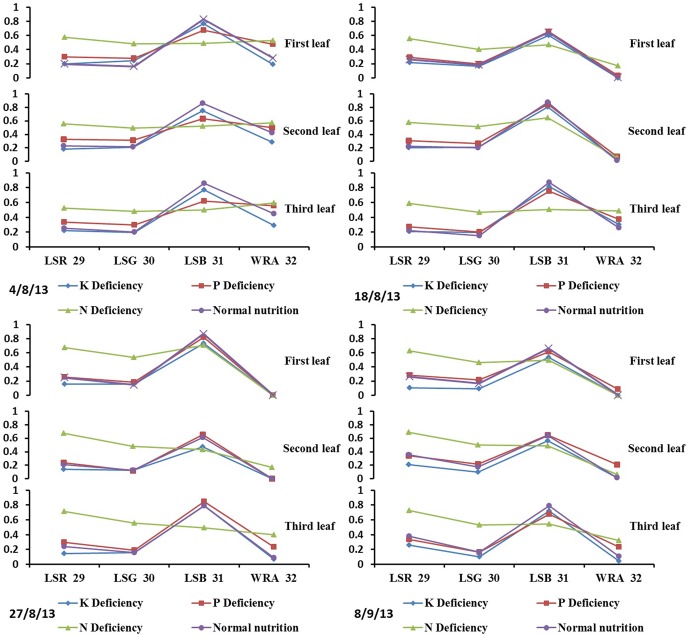
Color features of the rice sheath under different nutrition deficiencies.

**Figure 6:**
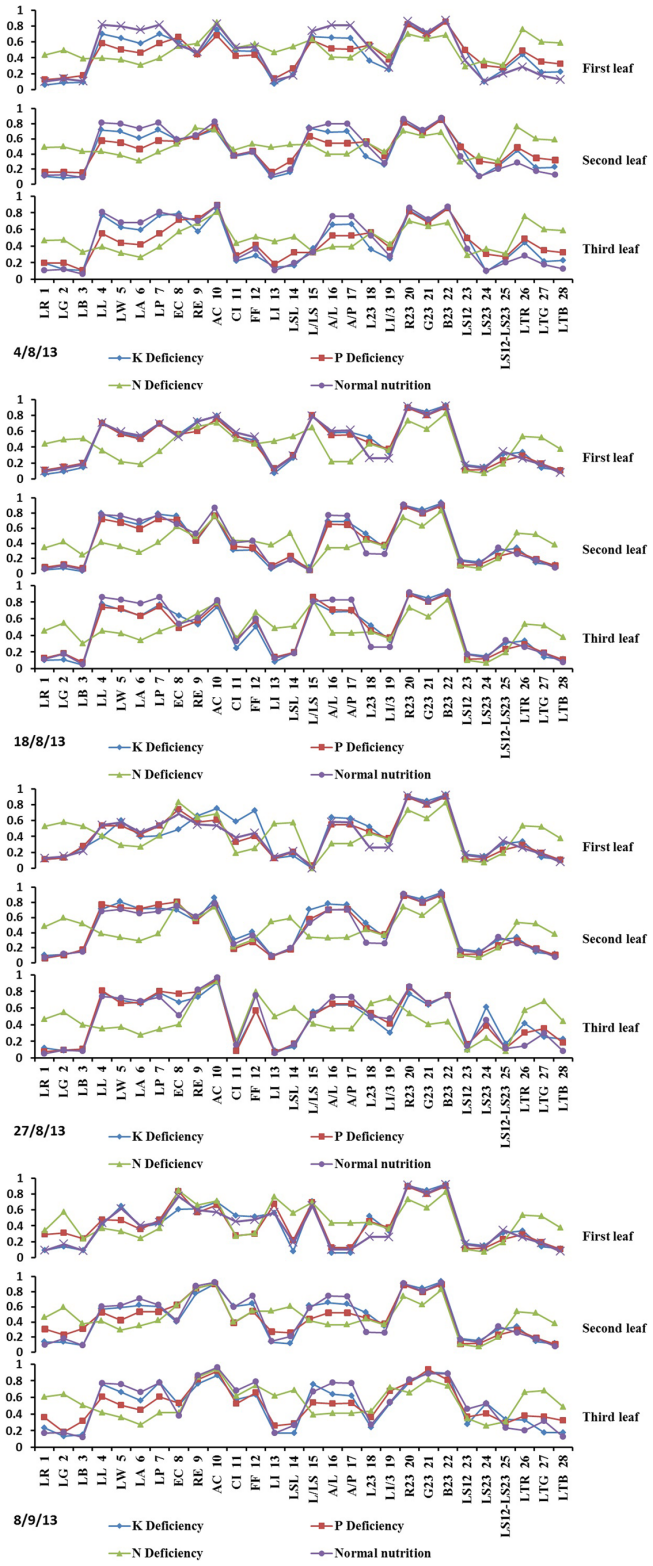
Rice characteristics under different types of nutrition stress.

As shown in Fig. 5 and Fig. 6 the color and shape characteristics of the leaf and leaf sheath were different under different nutrition deficiencies. The nutrition deficiency category can be identified from the characteristics, but using many characteristics can result in redundant information, which increases the number of calculations and influences the identification accuracy. However, it is difficult to recognize stress with so many sensitive characteristics. Therefore, to quickly diagnose nutrition deficiencies, it is necessary to choose the optimal set of characteristics using an effective feature-selection method.

The support vector feature selection (SVFS) method was used to select the optimal characteristic set to reduce the calculation burden and remove redundant information. This method makes full use of the advantage of SVM, namely, its generalizability from a small training sample. Additionally, this method can improve the operational efficiency and ensure a rapid and stable screening process. The optimal characteristic set has the maximum classification prediction ability. The method can remove the redundant characteristics of the subset by identifying high correlations between characteristics. Therefore, the optimal characteristics subset can represent the set of sensitive characteristics of the different types of nutrition statuses [16]–[19]. In this research, the SVFS screen characteristics subset in Libsvm-3.12 was used.

In the screening process, the rate of contribution to the identification was first estimated for each characteristic to sort and remove the unimportant characteristics until the screening process was complete. An SVFS measures the contribution of each feature to identification by disturbing the objective function of the SVM.

SVFS was used to select the optimal characteristics set from the twenty-eight characteristics extracted from the samples having NPK deficiencies and having normal nutrition levels. The essence of the screening is to select the optimal subsets from the original combination of characteristics to ensure accurate identification based on a minimum number of characteristics.

Fisher discriminant analysis can be used to determine which class a research object belongs to by observing and measuring the value of the variables. Discriminant analysis establishes a discriminant function by filtering the variables and by including information from those variables that can be used to determine the classification and characteristics of the object. The error rate can be minimized using this method, and its common formulation is
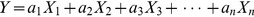
(2)


Here, y is the discriminant value; x_1_, x_2_, x_3_…, x_n_ are variables that reflect the characteristics of the research object; and a_1_, a_2_, a_3_…, a_n_ are discriminant coefficients [20], [21].

## Results and Discussion

The symptoms of NPK deficiencies in a rice leaf were markedly different between the four growth stages. Under N deficiency, the old leaves, and sometimes all leaves, become light green and chlorotic at the tip. Except for young leaves, which are greener, deficient leaves are narrow, short, and lemon-yellowish. Under P deficiency, the plants are stunted and dark green with erect leaves. The leaves are narrow, short, very erect, and ‘dirty’ dark green, and the stems are thin and spindly. Young leaves may appear to be healthy, but older leaves turn brown and die. Under K deficiency, the plants are dark green, and yellowish-brown leaf margins, or dark-brown, necrotic spots first appear on the tips of older leaves. Under severe K deficiency, the leaf tips are yellowish-brown. Symptoms first appear on older leaves and subsequently along the leaf edge and, finally, on the leaf base. Older leaves change from yellow to brown, and if the deficiency is not corrected, discoloration gradually appears on younger leaves [1].

In this research, the color of the leaf sheath was introduced to first diagnose the nutrition status. Under P deficiency, red and purple colors may develop in the leaf sheathes if the variety has a tendency to produce anthocyanin. When suffering from N deficiency, rice stems appear light green, and older sheaths become lemon-yellowish. The base of the leaf sheath appears white under severe stress [14].

In this research, the optimal subsets of the characteristics represented the set of the most sensitive characteristics under different nutrition deficiencies. The results are shown in Table 4 (the numbers represent the characteristics).

**Table 4:** Selected feature subsets of rice leaves in different positions.

Growth Stage	Leaf Position	Subset
4/8/13	1^st^ leaf	1, 2, 4, 14, 15, 18, 19, 22, 24
	2^nd^ leaf	2, 4, 6, 13, 14, 16, 18, 19, 21, 25, 28
	3^rd^ leaf	2, 6, 7, 8, 10, 13, 17, 18, 19, 21, 22, 24, 26
18/8/13	1^st^ leaf	2, 6, 14, 16, 17, 18, 27, 28
	2^nd^ leaf	2, 4, 6, 7, 8, 14, 18, 20, 28
	3^rd^ leaf	2, 5, 8, 9, 10, 11, 17, 18, 19, 24, 26
27/8/13	1^st^ leaf	2, 3, 8, 13, 26, 27, 28
	2^nd^ leaf	2, 3, 7, 10, 20, 22, 26, 27, 28
	3^rd^ leaf	2, 3, 13, 16, 20, 21, 26, 27, 28
8/9/13	1^st^ leaf	1, 2, 8, 10, 12, 14, 19, 23, 26, 27
	2^nd^ leaf	2, 3, 10, 14, 16, 17, 19, 26, 27
	3^rd^ leaf	1, 2, 4, 14, 15, 18, 19, 22, 24
Universal characteristics		2, 4, 6, 13, 14, 15, 18, 19, 20, 26

Note: 4/8/13, 18/8/13, 27/8/13, and 8/9/13 indicate the 4 growth stages (August 4^th^, August 18^th^, August 27^th^, and September 8^th^, respectively).

As shown in Table 4, the optimal set of characteristics of the different leaf positions included the leaf color, leaf shape, and sheath length in every growth stage, which means that every nutrition deficiency can affect the 3 types of characteristics. Taking the leaf and leaf sheath samples of all growth stages together, 9 characteristics (LG, LW, AC, LSL, L23, L1/3, LTR, and LTG) were determined to be universal characteristics for all growth stages for the identification of nutrition deficiencies.

After screening the characteristics, a Fisher discriminant analysis was used to identify the nutrition status of the rice. N, P, K and Normal represented nitrogen deficiency, phosphorus deficiency, potassium deficiency and normal nutrition, respectively. In this research, the IBM SPSS statistics 20 software package was used for the analysis.

For every growth stage, 390 samples were used to build the model. The results are shown in Table 5. As shown in Table 5, the accuracy was lower for the overall identification of NPK deficiencies. In later testing, N deficiency was easily misjudged as K deficiency, which led to a reduced overall accuracy. To increase the model's identification accuracy, new characteristics specific to N deficiency were needed. Previous research reported that old rice leaves and stems become light green under N stress and that the leaf sheath base becomes white under severe N deficiency [14]. Thus, in the present study, the color characteristics of the leaf sheath were introduced to increase the N stress identification accuracy.

**Table 5:** Overall recognition accuracy.

Leaf position	Growth stages			
	4/8/13	18/8/13	27/8/13	8/9/13
1^st^ leaf	73.08%	70.77%	72.31%	71.54%
2^nd^ leaf	79.23%	73.08%	73.85%	74.62%
3^rd^ leaf	**80.77%**	**75.38%**	**77.69%**	**77.69%**

Note: the highest identification accuracy for every growth stage is in bold. 4/8/13, 18/8/13, 27/8/13, and 8/9/13 indicate the 4 growth stages.

After adding the four parameters, Fisher discriminant analysis was used to identify the nutrition deficiencies. The results in Table 6 show that the overall identification accuracy was greatly improved, which verifies the importance of using the leaf sheath in the diagnosis of nutrition deficiencies.

**Table 6:** Identification accuracy with the additional color characteristics of leaf sheath.

Leaf position	Growth stages			
	4/8/13	18/8/13	27/8/13	8/9/13
1^st^ leaf	80.77%	75.38%	90.00%	80.77%
2^nd^ leaf	80.00%	77.69%	87.69%	83.08%
3^rd^ leaf	**83.85%**	**79.23%**	**90.00%**	**84.62%**

Note: the highest identification accuracy for every growth stage is in bold. 4/8/13, 18/8/13, 27/8/13, and 8/9/13 indicate the 4 growth stages.

According to Table 6, the leaf position with the highest identification accuracy was the same for the four growth stages. The optimal leaf position was the third leaf, which coincided with NPK being very mobile in the rice. Under NPK deficiencies, the nutrients were translocated into the young leaves from the old, senescing leaves, and the symptoms first appeared on the older leaves. Further testing was conducted to discover additional details about the identification of NPK deficiencies. The results are shown in Tables 7 and 8. According to the results, Normal nutrition and N deficiency had higher identification accuracies for all growth stages compared with P and K deficiencies. As shown in Table 8, the probabilities of identification error for P and K deficiencies were higher for all growth stages.

**Table 7:** Identification accuracy of different nutrition statuses.

Growth stage	Normal	N	P	K
4/8/13	90.00%	95.00%	85.00%	72.50%
18/8/13	90.00%	95.00%	77.50%	62.50%
27/8/13	90.00%	100.00%	87.50%	82.50%
8/9/13	100.00%	97.50%	87.50%	65.00%

Note: 4/8/13, 18/8/13, 27/8/13, and 8/9/13 indicate the 4 growth stages.

**Table 8:** Identification accuracy for P and K deficiencies.

	Deficiency	N(Misjudgment)	P(Misjudgment)	K(Misjudgment)	Normal(Misjudgment)
4/8/13	K	2.50%	85.00%	2.50%	10.00%
	P	7.50%	2.50%	72.50%	17.50%
18/8/13	K	5.00%	77.50%	2.50%	15.00%
	P	7.50%	7.50%	62.50%	22.50%
27/8/13	K	0.00%	87.50%	5.00%	7.50%
	P	5.00%	2.50%	82.50%	10.00%
8/9/13	K	2.50%	87.50%	2.50%	7.50%
	P	7.50%	5.00%	65.00%	22.50%

Note: 4/8/13, 18/8/13, 27/8/13, and 8/9/13 indicate the 4 growth stages.

The universal characteristics were used to build the rule in the identification process, but the rule was not targeted to identify particular nutrition deficiencies. To improve the model's identification accuracy and to reduce the misjudgment of P and K deficiencies, hierarchical identification was used.

In every hierarchy, the identification rule was targeted at one nutrition deficiency; thus, different optimal characteristics were selected in different hierarchies (Table 9). SVFS was used to screen the optimal characteristic parameters for every identification hierarchy. The results are shown in Table 9. Putting all of the leaf and leaf sheath samples together, the specific universal characteristic set from every hierarchy that was suitable for all stages was screened for the targeted nutrition deficiency. In the results described above, normal nutrition had the highest identification accuracy. Therefore, the first hierarchy was the identification of Normal and Non-normal nutrition, the set was LSR, LSG, LSB, LSL, LTR, LTG, LA and LG. For the identification of N and PK deficiencies, the set was LSG, LSB, WRA, LB, and LG. For the identification of P and K deficiencies, the set was LG, LSL, A/L, LTG, L23, LSG, and LI.

**Table 9:** Subset of selected rice characteristics in every hierarchy.

Hierarchy	Identification type	Subset
1^st^ hierarchy	Normal and Non-normal(NPK)	2, 6, 14, 26, 27, 29, 30, 31
2^nd^ hierarchy	N and PK	2, 3, 30, 31, 32
3^rd^ hierarchy	P and K	2, 13, 14, 16, 24, 27, 30

In the hierarchical identification process, Normal and Non-normal were identified first; thus, the characteristics that appeared under Non-normal nutrition were screened. Under N deficiency, rice leaves become light green, and red and purple colors may develop in stems under P deficiency due to anthocyanin. Under K deficiency, leaf tips are yellowish-brown; thus, the characteristics screened in the first hierarchy were leaf, leaf tip and leaf sheath color. For the identification of N and PK deficiencies in the second hierarchy, the characteristics that mainly appeared under N deficiency were screened. Under N deficiency, rice leaves and sheaths become light green; thus, the selection of characteristics mainly focused on leaf and leaf sheath color. For the identification of P and K deficiencies in the third hierarchy, each specific characteristic of P and K deficiencies was selected for identification. Under P deficiency, leaves are narrow, very erect, and ‘dirty’ dark green, and leaf sheaths are red and purple; thus, LI, EC, LSR, etc. were selected. Under P and K deficiencies, the leaves become chlorotic at the tip, but the affected area is different. Therefore, the color of the leaf tip was also selected.

According to the screening results, the Fisher discriminant analysis established three targeted rules for the identification of NPK deficiencies. For the 2013 dataset, 1560 samples (4 growth stages) were used to build the model to identify NPK deficiencies. The identification accuracy is shown in Table 10. As shown in Table 10, the leaf positions with the highest identification accuracy were the same for the four growth stages. The optimal leaf position was the third leaf and was the same as the optimal leaf positions mentioned above. Under NPK deficiencies, nutrients were translocated into the young leaves from old, senescing leaves; thus, the symptoms first appeared on older leaves.

**Table 10:** Training accuracy using hierarchical identification.

Leaf position	Growth stage			
	4/8/13	18/8/13	27/8/13	8/9/13
1^st^ leaf	85.38%	73.85%	84.62%	80.00%
2^nd^ leaf	85.38%	83.85%	88.46%	83.08%
3^rd^ leaf	**86.15%**	**87.69%**	**90.00%**	**89.23%**

Note: the highest identification accuracy in every growth stage is in bold. 4/8/13, 18/8/13, 27/8/13, and 8/9/13 indicate the 4 growth stages.

Comparing Table 6 with Table 10 shows that using hierarchical identification can effectively improve the identification accuracy. Table 11 shows the identification accuracy of one nutrition deficiency (N, P, K, and Normal) when the overall identification accuracy was the highest. The identification accuracy of P and K deficiencies was improved in all growth stages (Table 11). To evaluate the hierarchical identification and to validate the three models, 780 samples of four growth stages (July 29^th^, August 13^th^, August 20^th^, and August 31^st^) collected in 2012 were used to validate the models. The results are shown in Table 12. The results show that hierarchical identification can be used to effectively identify NPK nutrition deficiencies, and the optimal leaf positions for identification conformed to the physiological characteristics of rice under the different nutrition deficiencies in all growth stages. As shown in Table 13, the validation accuracy for one nutrition deficiency (N, P, K, and Normal) was higher using hierarchical identification.

**Table 11:** Training accuracy for the identification of different nutrition deficiencies.

Growth stage	Normal	N	P	K
4/8/13	80.00%	95.00%	80.00%	85.00%
18/8/13	80.00%	100.00%	82.50%	82.50%
27/8/13	90.00%	100.00%	80.00%	90.00%
8/9/13	80.00%	95.00%	85.00%	90.00%

Note: 4/8/13, 18/8/13, 27/8/13, and 8/9/13 indicate the 4 growth stages.

**Table 12:** Validation accuracy using hierarchical identification.

Leaf position	Growth stage			
	29/7/12	13/8/12	20/8/12	31/8/12
1^st^ leaf	76.92%	61.54%	80.00%	78.46%
2^nd^ leaf	81.54%	67.69%	83.08%	83.08%
3^rd^ leaf	**83.08%**	**83.08%**	**89.23%**	**90.77%**

Note: the highest identification accuracy for every growth stage is in bold. 29/7/12, 13/8/12, 20/8/12, and 31/8/12 indicate the 4 growth stages.

**Table 13:** Validation accuracy for the identification of different nutrition deficiencies.

Growth stage	Normal	N	P	K
29/7/12	80.00%	80.00%	90.00%	80.00%
13/8/12	60.00%	90.00%	85.00%	80.00%
20/8/12	80.00%	95.00%	90.00%	85.00%
31/8/12	100.00%	85.00%	100.00%	85.00%

Note: 29/7/2012, 13/8/2012, 20/8/2012, and 31/8/2012 indicate the 4 growth stages.

## Conclusions

This paper takes the three most anterior leaves and sheaths of rice under different NPK nutrition conditions as the object of research. Under laboratory conditions, the color and shape parameters were acquired from the scanned images of rice leaves and sheaths. Then, SVFS was used to select the optimal characteristics set for the identification of NPK deficiencies, and Fisher discriminant analysis was used to build the diagnosis model.

Compared with traditional methods, hierarchical identification can be used to effectively build the targeted identification rules for each nutrition deficiency. Finally, to improve the accuracy of the identification, this research introduced hierarchical identification. In this research, the identification process was divided into three hierarchies. First, the normal and non-normal (NPK deficiencies) samples were identified. In this hierarchy the selected characteristics were LSR, LSG, LSB, LSL, LTR, LTG, LA and LG. In the second hierarchy, N deficiency and PK deficiencies were identified. In this hierarchy the selected characteristics were LSG, LSB, WRA, LB, and LG. Finally, P deficiency and K deficiency were identified. In this hierarchy the selected characteristics were LG, LSL, A/L, LTG, L23, LSG, and LI.

The result showed that hierarchical identification can be used to effectively improve the accuracy of identification (86.15, 87.69, 90.00 and 89.23% for the 4 growth stages). Data representing different years were used for validation, and the validation accuracies were 83.08, 83.08, 89.23 and 90.77% for the 4 growth stages.

The study provides evidence for the quick diagnosis of rice nutrient status, which makes it possible to accurately identify rice NPK deficiencies with scanning technology. Other crops, such as maize and wheat, suffering nutrition deficiencies usually exhibit some special symptoms on the leaves and sheaths, and this method can be used to diagnose their nutrition level status. Therefore, the technology introduced in the study has application value and development potential.

## References

[1] Armstrong DL (2002) Nutrient Deficiency Symptoms in Rice. Better Crops International 16 , Special Supplement: 23–25.

[2] Thomas JR, Oerther GF (1972) Estimating nitrogen content of sweet pepper leaves by reflectance measurements. AGRON J 64(1):11–13.

[3] Everitt JH, Pettit RD, Alaniz MA (1987) Remote Sensing of Broom Snakeweed (Gutierreziasarothrae) and Spiny Aster (Aster spinosus). WEED SCI 35(2):295–302.

[4] Shibayama M, Akiyama T (1989) Seasonal visible, near-infrared and mid-infrared spectra of rice canopies in relation to LAI and above-ground dry phytomass. REMOTE SENS ENVIRON 27(2):119–127.

[5] Milton NM, Eiswerth BA, Ager CM (1991) Effect of phosphorus deficiency on spectral reflectance and morphology of soybean plants. REMOTE SENS ENVIRON 36(2):121–127.

[6] Shibayama M, Akiyama T (1991) Estimating grain yield of maturing rice canopies using high spectral resolution reflectance measurements. REMOTE SENS ENVIRON T 36(1):45–53.

[7] Shibayama M, Takahashi W, Morinaga S, Akiyama T (1993) Canopy water deficit detection in paddy rice using a high resolution field spectroradiometer. REMOTE SENS ENVIRON 45(2):117–126.

[8] Lin FF, Ding XD, Fu ZP, DengG JS, Shen ZQ (2009) Application of Mutual Information to Variable Selection in Diagnosis of Phosphorus Nutrition in Rice. SPECTROSC SPECT ANAL 9:2467–2470. 19950654

[9] Jia LL, Fan MS, Zhang FS, Chen XP, Lu SH, et al (2009) Nitrogen Status Diagnosis of Rice by Using a Digital Camera.SPECTROSC SPECT ANAL. 29(8):2176–2179. 19839333

[10] Shi YY, Deng JS, Chen LS, Zhang DY, Ding XD, et al (2009) Leaf Characteristics Extraction of Rice under Potassium Stress Based on Static Scan and Spectral Segmentation Technique. SPECTROSC SPECT ANAL 29(7):1745–1748. 20302117

[11] Chen LS, Zhang SJ, Wang K, Shen ZQ, Deng JS (2013) Identifying of rice phosphorus stress based on machine vision technology. LIFE SCI J. 10(2):2655–2663.

[12] Chen LS, Sun YY, Wang K (2014) Identifying of rice nitrogen stress based on machine vision and multi-scale information extraction. SENS LETT. 12:824–830.

[13] Chen LS, Wang K (2014) Diagnosing of rice nitrogen stress based on static scanning technology and image information extraction. J SOIL SCI PLANT NUT. 14(2):382–393.

[14] Li WM (2012) Crop symptoms under nutrition stress. Qinghai Agro-Technology Extension 2:44–45.

[15] Tkalcic M, Tasic JF (2003) Colour spaces: perceptual, historical and applicational background. Eurocon.

[16] Weston J, Mukherjee S, Chapelle O, Pontil M, Poggio T, et al (2000) Feature selection for SVMs. NIPS 12:68–674.

[17] Fung GM, Mangasarian OL (2004) A feature selection Newton method for support vector machine classification. COMPUT OPTIM APPL 28(2):185–202.

[18] Li GZ, Wang M, ZengHJ (2004) An Introduction to Support Vector Machines and other Kernel Based Learning Methods. Bejing: Publishing House of Electronics Industry Press.

[19] Glenn MF, Mangasarian OL (2004) A Feature Selection Newton Method for Support Vector Machine Classification. COMPUT OPTIM APPL 2(28):185–202.

[20] Zhao F, Zhang JY, Liang JL (2007) A Fast Algorithm about Kernel Fisher Discriminant Analysis. J ELECTRON INFORM TECHNOL 7(29):1731–1734.

[21] Liu QS, Lu HQ, Ma SD (2004) Improving kernel Fisher discriminant analysis for face recognition. Circuits and Systems for Video Technology, IEEE Transactions on 14(1):42–49.

